# Dispersal of microbes from grassland fire smoke to soils

**DOI:** 10.1093/ismejo/wrae203

**Published:** 2024-10-15

**Authors:** Adam J Ellington, Kendra Walters, Brent C Christner, Sam Fox, Krista Bonfantine, Cassie Walker, Phinehas Lampman, David C Vuono, Michael Strickland, Katie Lambert, Leda N Kobziar

**Affiliations:** Department of Microbiology and Cell Science, Institute of Food and Agricultural Science, University of Florida, P.O. Box 110700 Gainesville, FL 32611, United States; Department of Forest, Rangeland, and Fire Sciences, University of Idaho, 1031 N. Academic Way, Coeur d’Alene, ID 83814, United States; Department of Microbiology and Cell Science, Institute of Food and Agricultural Science, University of Florida, P.O. Box 110700 Gainesville, FL 32611, United States; Department of Forest, Rangeland, and Fire Sciences, University of Idaho, 1031 N. Academic Way, Coeur d’Alene, ID 83814, United States; Department of Forest, Rangeland, and Fire Sciences, University of Idaho, 1031 N. Academic Way, Coeur d’Alene, ID 83814, United States; Department of Biology, Brigham Young University – Idaho, 525 S Center St., Rexburg, ID 83460, United States; Department of Forest, Rangeland, and Fire Sciences, University of Idaho, 1031 N. Academic Way, Coeur d’Alene, ID 83814, United States; Department of Civil and Environmental Engineering, Colorado School of Mines, 1500 Illinois St., Golden, CO 80401, United States; Department of Soil and Water Systems, University of Idaho, 875 Perimeter Drive, Moscow, ID 83844, United States; Department of Soil and Water Systems, University of Idaho, 875 Perimeter Drive, Moscow, ID 83844, United States; Department of Forest, Rangeland, and Fire Sciences, University of Idaho, 1031 N. Academic Way, Coeur d’Alene, ID 83814, United States

**Keywords:** wildfire, microbial ecology, prairie, smoke transport, soil respiration, biodiversity, fire ecology

## Abstract

Wildland fire is increasingly recognized as a driver of bioaerosol emissions, but the effects that smoke-emitted microbes have on the diversity and community assembly patterns of the habitats where they are deposited remain unknown. In this study, we examined whether microbes aerosolized by biomass burning smoke detectably impact the composition and function of soil sinks using lab-based mesocosm experiments. Soils either containing the native microbial community or presterilized by γ-irradiation were inundated with various doses of smoke from native tallgrass prairie grasses. Smoke-inundated, γ-irradiated soils exhibited significantly higher respiration rates than both smoke-inundated, native soils and γ-irradiated soils exposed to ambient air only. Microbial communities in γ-irradiated soils were significantly different between smoke-treated and control soils, which supports the hypothesis that wildland fire smoke can act as a dispersal agent. Community compositions differed based on smoke dose, incubation time, and soil type. Concentrations of phosphate and microbial biomass carbon and nitrogen together with pH were significant predictors of community composition. Source tracking analysis attributed smoke as contributing nearly 30% of the taxa found in smoke-inundated, γ-irradiated soils, suggesting smoke may play a role in the recovery of microbial communities in similar damaged soils. Our findings demonstrate that short-distance microbial dispersal by biomass burning smoke can influence the assembly processes of microbial communities in soils and has implications for a broad range of subjects including agriculture, restoration, plant disease, and biodiversity.

## Introduction

Microbial aerosolization and dispersal patterns are of interest across a diverse array of disciplines including agriculture [1–3], human health [4–6], meteorology [7–9], and biogeography [10]. Wildland fires (wildfires and prescribed fires) are well understood to have terrestrial biophysical and atmospheric physicochemical impacts [11], but the smoke they generate has only recently been explored as a driver of bioaerosol emissions and dissemination [12–15]. Smoke’s role as a vector for bioaerosol transport shifts existing paradigms of a wildland fire’s perimeter of biological impact [12, 16, 17]. Existing literature is limited in examination of smoke’s impacts on microbial communities and processes. Overall, results range from beneficial to detrimental on diversity, biomass, and functioning, but prior studies do not approach the question with the perspective of smoke as a dispersal agent for viable microbes [18–22]. As climate change continues to create ideal conditions for more severe and longer-burning wildfires in many regions of the world [23, 24], a complete understanding of the ecological and societal effects of microbes aerosolized by biomass burning is of paramount importance.

Biomass burning consumes 8800 Tg of vegetative materials annually around the globe [25], with a yet-unknown percentage comprised of living microorganisms [14]. Recent studies have demonstrated that the majority of microbial cells (>70%) emitted by wildland fire remain viable in the local smoke plume [13, 15]. Likewise, global air pollution studies have found significant differences in indicators of increased microbial metabolic activity during periods when biomass burning smoke concentrations are high compared to non-smoke periods [26, 27]. Yet, rates of long-term survival, deposition, and colonization of sink habitats by smoke-emitted microbes remain unknown and have direct relevance to understanding the full extent of the impact of wildland fire on community ecology.

Microbial community assembly processes range across the stochastic-deterministic spectrum [28]. Stochastic processes include probabilistic dispersal, random speciation/extinction, and ecological drift, whereas deterministic processes include environmental filtering by abiotic factors and biotic interactions (e.g., competition, synergy) [18]. Although some studies suggest that stochastic immigration processes have a stronger influence on microbial community assembly in soils [29, 30], fire has been shown to increase neutral assembly processes in soil microbial communities [31]. However, the influence of smoke-transported microbes has yet to be accounted for in current theories of microbial biogeographical relationships, diversity, and community assembly patterns. In the many global ecosystems where fire has shaped the evolution and distribution of life [11, 16, 32, 33], understanding whether smoke-dispersal of microbes has a discernable and predictable effect on sink habitat communities could transform current understanding of microbial diversity patterns across space and time.

**Figure 1:**
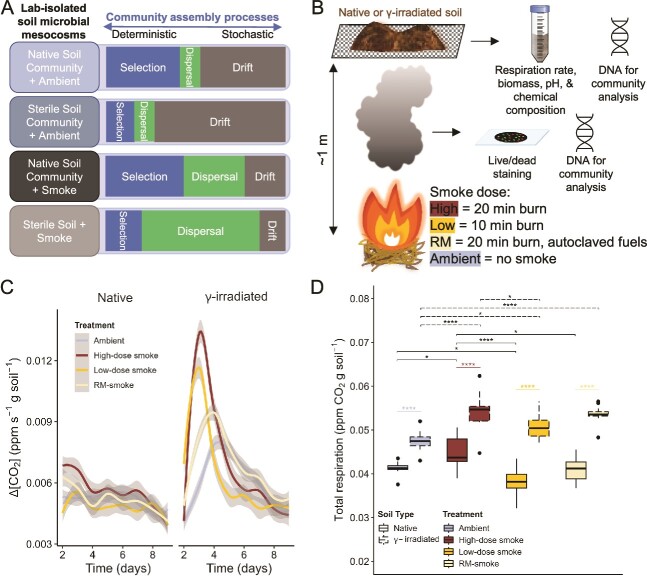
**Effect of smoke inundation on soil respiration.** (**A**) Conceptual diagram depicting how the experimental design reflects shifts in the relative influence of soil community assembly processes in lab-isolated mesocosms, comparing native soils exposed to ambient conditions (i.e., no smoke); native soils inoculated with smoke; and pre-sterilized soils with smoke. Terms as defined in Vellend et al. (2010). (**B**) Schematic depiction of soil inoculation procedure and subsequent analyses. See sections 2.2–2.5 for details. (**C**) Soil respiration rates measured as Δ[CO2] (ppm s^-1^ g soil^-1^) for the first 8 days of incubation. Grey area around each line represents 95% confidence interval. (**D**) Sum of the respiration rates for the first 8 days of incubation. Boxes represent the interquartile range, the middle horizontal line is the median, and the whiskers represent minimum and maximum values. Outliers are 1.5 interquartile ranges below the first quartile and above the third quartile. Asterisks represent significance (^*^ = *P <* 0.05; ^*^^*^^*^^*^ = *P <* 0.0001; Tukey’s post-test).

In this study, we examined microbial community assembly processes and source/sink relationships in controlled, lab-based soil mesocosms inundated with biomass burning smoke. High severity fires may damage surface soils, with previous studies reporting significantly reduced microbial biomass [34, 35], CO_2_ respiration [34, 36], fungal abundance [36], and shifts in bacterial community composition [35] compared to low severity fires [37]. Accordingly, untreated (“native”) and gamma-irradiated soil environments were used in our experiments to contrast the effects of smoke inundation in unburned/low disturbance scenarios and in highly disturbed soils, respectively. Control soils and soils receiving varying doses of smoke were compared to explore the relative contribution of selection, dispersal, and drift mechanisms on community assembly. Smoke from autoclaved fuels was also included in the experimental design with the goal of distinguishing the effects of physicochemical smoke constituents versus transported viable microbes. We hypothesized that (i) Smoke-inundated soil communities would differ between native and sterilized soils consistent with differences in smoke’s impact on community assembly processes between the two soil habitats (Fig. 1A); (ii) Smoke-inoculated, sterilized soils would have greater similarity to smoke assemblages than native soils, higher diversity than non-inundated sterilized soils or those inoculated with smoke from autoclaved fuels, and lower genetic diversity than native soils (i.e., founder effect); (iii) The carbon efflux signal resulting from smoke inundation would be more easily detected in sterilized than in native soils and higher “doses” of smoke from non-autoclaved fuels would result in increased soil respiration and impacts on soil physicochemical properties. The results of this study indicate that microbial dispersal by biomass burning smoke can influence the assembly processes of microbial communities in soils and suggest that smoke promotes connections between non-contiguous soil sources and sinks via bioaerosol dispersal.

## Materials and methods

### Site description and collection of organic fuels and soils

The Konza Prairie Biological Station (KPBS) in the Flint Hills of northeastern Kansas, USA consists of ~35 km^2^ of prairie dominated by C4 grasses (e.g., *Andropogon gerardii*, *Schizachyrium scoparium*, *Sorghastrum nutans*, *Panicum virgatum*) with Benfield-Florence complex silty clay loams. KPBS is divided into ~50 watershed units, each subjected to prescribed burning at 1-, 2-, 3-, 4-, or 20-year intervals. A total of 60 soil cores (~5 cm depth) were collected from plot K2A (burned biennially; last burned 1 year prior to sampling) of KPBS using a trowel presterilized with 70% ethanol. The thin organic layer (O-horizon) of the soil was not included in the sample, whereas the surface layer (A-horizon) was placed into ventilated plastic collection bags on ice. Fuels (~95% tallgrass species, ~5% forbs or woody shrubs) were collected from randomized plots in K1A (burned annually since at least 1979), K20A (burned annually since 2012), and K1B (burned annually since at least 1979) and stored on ice. Soil and fuel samples were shipped on ice to the University of Idaho in Couer d'Alene, ID, USA and subsequently stored in the dark at 4°C. Soils were sieved to 2 mm prior to inundation and incubation.

Approximately 2 L of the collected soils were exposed to ^60^Co γ-radiation treatment at the Radiation Science and Engineering Center at Pennsylvania State University. The soil was irradiated for 33 h and 40 min, corresponding to a dose of 40 kGy as determined by radiochromic film dosimetry. Irradiated soils were stored in sealed sterile bags in the dark at 4°C for two weeks prior to the experiment to allow any post-radiation effects on microbial activity to stabilize. The water holding capacity (WHC) for both the native and γ-irradiated soil was determined as described previously [38] and, following the smoke or ambient air treatments, soils were maintained near field capacity (65% WHC) with regular additions of sterile dH_2_O [38].

### Smoke inundation of soils and aerosol sampling

The University of Idaho IFIRE Combustion Lab was used to conduct three burns: low-dose smoke, high-dose smoke, and smoke from fuels autoclaved at 121°C for 1 h (reduced microbial smoke; “RM-smoke”). The lab is equipped with a high-volume exhaust hood and all inlets were sealed with HEPA filters to limit introduction of outside aerosols during the experiment. Dosages are reflected in significantly different measured levels of particulate matter (PM) produced (Table 1). Chilled fuels were thawed overnight at 25°C prior to conducting the burns. “Low-dose smoke” reflected 10 min of continuous feeding of biomass into the fire with exposure time-averaged PM_10_ of 376 μg m^−3^ and PM_2.5_ of 391 μg m^−3^, and “High-dose smoke” reflected 20 min of continuous fuel additions to the fire with exposure time-averaged PM_10_ of 2181 μg m^−3^ and PM_2.5_ of 2227 μg m^−3^ (Fig. 1B).

**Table 1:** Bioaerosol, particulate matter (PM) emissions, and environmental data during each sampling period.

	**Ambient**	**High-dose Smoke**	**Low-dose Smoke**	**RM-Smoke**
**PM**_**10**_ (**μg m**^**−3**^)	7.44	2180.96	376.15	1372.18
	± 0.10	± 127.61	± 21.50	± 121.68
**PM**_**2.5**_ (**μg m**^**−3**^)	5.3	2226.77	391.18	1430.79
	± 0.05	± 115.56	± 27.82	± 122.14
**PM**_**1.0**_ (**μg m**^**−3**^)	3.78	260.8	182.54	180.38
	± 0.06	± 8.19	± 7.25	± 7.33
**Cell Counts (cells m** ^**−3**^)				
*Total Cells*	9.6 × 10^4^	2.01 × 10^5^	1.10 × 10^5^	1.15 × 10^5^
*Live Cells*	7.96 × 10^4^	1.83 × 10^5^	9.12 × 10^4^	9.58 × 10^4^
*Dead Cells*	1.64 × 10^4^	1.81 × 10^4^	1.89 × 10^4^	1.95 × 10^4^
*Total Spores*	9.37 × 10^1^	1.31 × 10^3^	9.38 × 10^2^	9.38 × 10^1^
**Temp (°C)**	15.19	26.97	18.65	21.13
	± 0.02	± 0.37	± 0.07	± 0.40
**RH (%)**	45.74	29.98	39.69	37.07
	± 0.06	± 0.46	± 0.11	± 0.53

Average ± SEM reported. RH, relative humidity.

An aluminum foil baking pan on a combustion table was used to contain the fuels ~1 m under a metal screen platform, to support and act as a spark arrestor for four sterilized silk screens holding thin (<0.5 cm thick) layers of native or γ-irradiated soils (two screens each with ~110 g of soil). Slightly above the metal screen platform, two Leland Legacy (SKC Inc., Eighty-Four, PA, USA) compensating programmable vacuum pumps were set up to sample the smoke’s microbial aerosols using a BioStage Impactor and a pair of Button Personal Aerosol Samplers (both from SKC, Inc., Eighty-Four, PA). Two replicate PurpleAir PA-II-SDs (PurpleAir, Inc.) were employed near the Leland pumps to measure PM concentration and collect meteorological data. The entire setup was positioned under the combustion room exhaust hood to pull the smoke directly through the soil and sampling devices. The combustion room was sterilized using 70% ethanol sprayed throughout the room and on all surfaces, along with Lysol disinfectant spray. Prior to any fuel combustion, ambient air was sampled for 20 min. Fuels were added systematically throughout each burn sampling period to achieve the desired smoke dosages.

### Cell counts

One of the two paired 25 mm PTFE filters in the Button samplers from each sampling period was stained for cell enumeration and viability. Samples were shaken at a low speed using a vortexer for 15 min in a 50 ml conical tube with 30 ml of 1X phosphate buffered saline (PBS). After shaking, the PBS solution was dispersed in 10 ml aliquots into a 3-tower vacuum manifold system and filtered onto black 0.2 μm polycarbonate filters (Millipore Sigma). Towers were rinsed with 10 ml of a 1X Tris/borate/EDTA (TBE) solution to aid in rinsing any cells that may have attached to the tower wall. Cells on two of the filters were stained with a 1:1 ratio of Syto9:propidium iodide, whereas cells on the third filter were stained with 25 μl Calcofluor White and one drop of potassium hydroxide. Towers were then covered in aluminum foil and incubated in the dark for 15 min. After the incubation period, the staining solutions were removed by applying vacuum pressure and the filters were rinsed with 10 ml of 1X TBE. Filters were then removed from the filter holders and mounted on glass microscope slides with 5 μl of antifade solution (1:1 of PBS and glycerol with 0.1 g of phenylenediamine) added both underneath and on top of the filter surface. Cover slips were applied to the slides and sealed with an ethyl acetate-based sealant (i.e., quick drying clear nail polish). Stained and fixed samples were stored in the dark at −20°C until further analyses were conducted. Using epifluorescence microscopy, fluorescing cells (Syto9: propidium iodide) or spores (Calcofluor White) were enumerated using forty random fields of view at 100x or 10x magnification for bacterial and fungal counts, respectively. These values were then averaged to derive a total cell count for each sample per unit volume of air or smoke sampled. Total bacterial cells were the sum of cells fluorescing green from Syto9 stain (viable cells; excitation/emission maxima 480/500 nm) plus cells fluorescing red from propidium iodide stain (dead cells; excitation/emission maxima 490/645 nm) as previously described [13].

### Determination of respiration rates, biomass, and pH

Following each burn, soils were transferred from the silk screens into sterile glass jars covered with 0.22 μm nylon mesh. In a sterile biosafety cabinet, the soils were brought to 65% WHC with sterile dH_2_O and kept at room temperature overnight. Soil from each treatment was distributed into twelve 50 ml conical tubes containing ~13 g each, covered with nylon filters, and incubated in a 25°C water bath in the dark for 35 days. Throughout the incubation period, soil samples were maintained at 65% WHC using sterile dH_2_O. Starting two days after treatment to allow for settling time in the incubation tubes, CO_2_ production of each soil sample was measured every 1–4 days using an LI-870 gas analyzer (LI-COR Biosciences, Lincoln, NE, USA). CO _2_ concentration was recorded over 2–4 min per sample, and soil respiration rate was calculated as Δ[CO_2_] (ppm s^−1^ g soil^−1^). CO_2_ rate measurements for empty vial controls were taken at each timepoint and subtracted from each sample. Six replicates of “control” soils for both native and γ-irradiated soils were not subjected to smoke or ambient air in the combustion lab, and instead remained in sterile containers in a − 80°C freezer until DNA extraction.

Ten days post-inundation, microbial biomass C and N was estimated using a modified chloroform fumigation-extraction method as described previously [39, 40]. Biomass C and N pools were estimated as the flush of dissolved organic C (DOC) or total N, respectively, following fumigation with ethanol-free chloroform. DOC, total N, and microbial biomass C and N were determined from the liquid extracts via a total organic carbon (TOC) / TN analyzer (Shimadzu, Columbia, MD, USA). Raw values are reported; no correction factors are used. Soil pH (1:1, soil:H2O by volume) was measured with a benchtop pH meter 35 days post-inundation. Ammonium levels were determined using the phenol-hypochlorite reaction as previously described [41], nitrates were quantified using a single reagent spectrophotometric method [42], and inorganic phosphorus was determined using previously published methods [43].

### DNA extraction, amplification, and sequencing

For the soil samples, total genomic DNA was extracted from ~0.25 g soil using the DNeasy Powersoil Pro Kit (Qiagen, Hilden, Germany) according to the manufacturer’s recommended protocol. Samples were taken before inundation (t = 0), 10 days post-inundation (t = 1), and 35 days post-inundation (t = 2). Extracted DNA was quantified with a ND2000 spectrophotometer (NanoDrop Technology, Wilmington, Delaware) and standardized to 10 ng/μl prior to PCR amplification. For the fuel samples, total genomic DNA was extracted from ~0.25 g fuels using the ZymoBIOMICS DNA Miniprep Kit (Zymo Research, Irvine, California, USA) following the manufacturer’s recommended protocol with slight modifications detailed in Supplemental Information. For the air and smoke samples, total genomic DNA was extracted from one of the two paired 25 mm PTFE filters in the Button sampling device using the ZymoBIOMICS Microprep Kit (Zymo Research, Irvine, California, USA) following a modified protocol detailed in Supplemental Information.

### Bioinformatics analysis

Amplicons were trimmed and filtered using the DADA2 (v.1.12.1) pipeline [44] in the R environment (v.3.6.0/v.4.2.1) as detailed in Supplemental Information. Data analysis and visualization were performed in the R environment using microbiome-specific packages (phyloseq, microbiome, and vegan), as well as custom R scripts. Samples containing <1000 reads after quality filtering and preprocessing were removed from subsequent analyses. Alpha diversity was assessed using the observed richness, Shannon, and Inverse Simpson diversity indices. Microbial community composition was visualized using non-metric, multidimensional scaling (NMDS) plots. FEAST [45] was used to estimate the relative contribution of source microbiomes to sink community composition for the two source/sink scenarios: fuel sources to smoke sinks and smoke sources to soil sinks. The ambient air samples were considered source environments in both scenarios. All fuel, smoke, and ambient air samples were merged respectively prior to analysis with FEAST, and the native and γ-irradiated soil samples were merged respectively based on the timepoint of sampling. Statistical analysis details are included in the Supplemental Information.

## Results

### Bioaerosol/PM emissions and environmental data

Average temperature, relative humidity (RH), and concentrations of PM_10_, PM_2.5_, and PM_1.0_ all differed significantly among treatment types (Kruskal-Wallis, *P <* 0.0001; Table 1). However, multiple pairwise comparisons using Dunn’s test revealed that PM_1.0_ concentrations and temperature were not significantly different between the low-dose and RM-smoke treatments (*P* > 0.05). Total and live cell counts were approximately twice as high in the high-dose smoke treatment than any other treatment, whereas the low dose and RM-smoke treatments exhibited similar counts (Table 1). Total spore counts were 1.5x higher in the high-dose smoke treatment compared to the low-dose, and 14x higher when compared to the RM-smoke and ambient control (Table 1).

### Cellular respiration rates in smoke inundated soils

Soil respiration rates were highest in the first eight days of post-incubation measurements, with peak rates observed between days two through four for all samples (Fig. 1C) and returning to near basal levels from approximately Day 9 through the remainder of the 32-day incubation (Supplementary Fig. A1A). Total respiration in the first eight days of measurement was significantly higher in γ-irradiated soils than in native soils (ANOVA, *P <* 0.0001; Fig. 1D). There were also significant differences in total respiration among treatment groups (*P <* 0.0001) that depended on the soil type, indicating a significant interaction between the two factors (*P <* 0.001). Pairwise comparisons using Tukey’s post-hoc test showed that within the γ-irradiated soils, average total respiration after inundation with high-dose, low-dose, and RM-smoke (5.4 ± 0.13 × 10^−2^ ppm CO_2_ g soil^−1^) were all significantly higher (*P <* 0.05) than for the ambient control. For the native soils, average total respiration after inundation with high-dose and RM-smoke was not significantly different than that of the ambient control but was significantly lower than the control after inundation with low-dose smoke (*P <* 0.05). Total respiration was significantly higher in γ-irradiated soil than native soil for smoke-treated samples but not for ambient controls. No significant differences in total respiration were observed between treatments when summed across the entire 32-day incubation (*P <* 0.05; Supplementary Fig. A1B).

### Microbial biomass and chemical composition of soils

**Figure 2:**
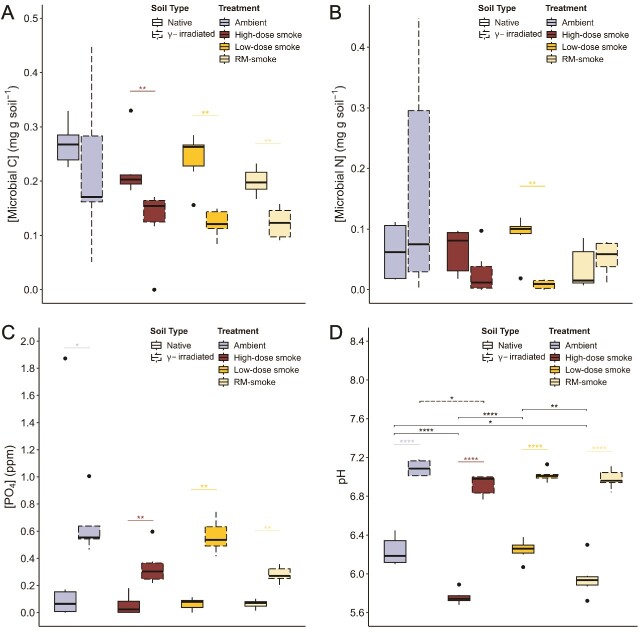
**Effect of smoke inundation on the chemical composition and pH of soils.** Measurements of (**A**) biomass C, (**B**) biomass N, and (**C**) phosphate concentrations for samples taken 10 days post-inundation and (**D**) pH for samples taken 35 days post-inundation. Boxes represent the interquartile range, the middle horizontal line is the median, and the whiskers represent minimum and maximum values. Outliers are 1.5 interquartile ranges below the first quartile and above the third quartile. Asterisks represent significance (^*^ = *P <* 0.05; ^*^^*^ = *P <* 0.01, ^*^^*^^*^^*^ = *P <* 0.0001; Dunn’s test [A-C]; Tukey’s post-test [D]).

Soil microbial biomass C in samples measured 10 days post-inundation was significantly reduced in smoke-inundated, γ-irradiated soils compared to smoke-inundated, native soils (*P <* 0.01; Fig. 2A). Microbial biomass N did not differ significantly between soil types, except when treated with low-dose smoke, where γ-irradiated soils had significantly less biomass N than native soils (*P <* 0.01; Fig. 2B). On the contrary, TOC concentrations exhibited the opposite trend, with γ-irradiated soils containing significantly higher TOC than native soils at all treatment levels (*P <* 0.01; Supplementary Fig. A2A). TN concentrations were significantly higher in the γ-irradiated soils than the native soils for the ambient and RM-smoke treatments only (*P <* 0.05; Supplementary Fig. A2B).

Phosphate concentrations were significantly higher in γ-irradiated soils than native soils for all treatments (*P <* 0.01; Fig. 2C). Ammonium concentrations were not significantly different between soil types (Supplementary Fig. A2C). Within the native soils, samples treated with RM-smoke contained significantly more ammonium than ambient controls (*P <* 0.05), but no other significant differences between treatments were observed (Supplementary Fig. A2C). No significant differences were detected in nitrate concentrations regardless of soil type or treatment condition (Supplementary Fig. A2D). Measurements of pH in samples taken 35 days post-inundation were significantly higher in the γ-irradiated soils than the native soils (*P <* 0.0001; Fig. 2D). Within the native soils, high-dose and RM-smoke treatments resulted in significantly lower pH than the ambient control (*P <* 0.0001 & 0.05, respectively) and low-dose smoke conditions (*P <* 0.0001 & 0.01, respectively; Fig. 2D).

### Microbial community composition in smoke inundated soils

**Figure 3:**
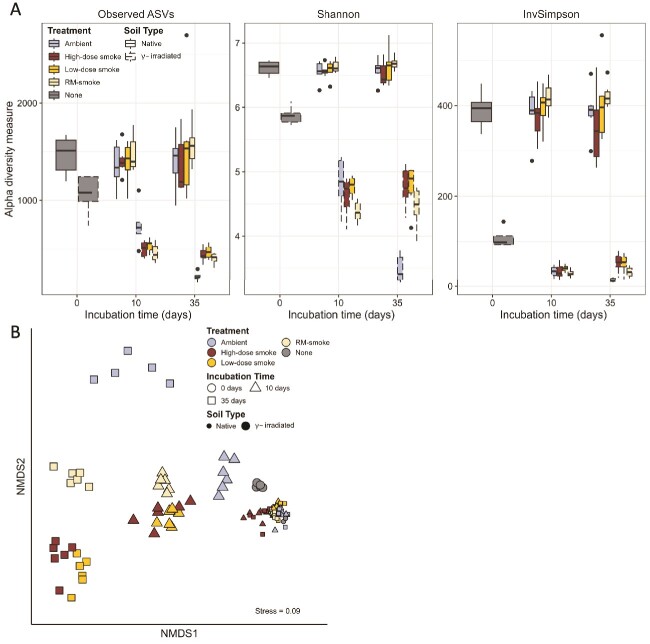
**Alpha diversity and composition of soil mesocosms.** (**A**) Observed ASVs, Shannon, and inverse Simpson diversity indices of taxa and (**B**) NMDS ordination of community composition by sample type for bacterial and archaeal (16S rRNA genes) communities in soil mesocosms.

Alpha diversity of the bacterial and archaeal communities was significantly higher in the native soils than the γ-irradiated soils (Welch’s ANOVA, *P <* 0.0001; Fig. 3A). Within the native soils, no difference in alpha diversity was observed between treatments or timepoints. However, in the γ-irradiated soils, alpha diversity at 10- and 35-days post-inundation was significantly lower under all treatment conditions than in the time zero (Treatment = “None”) controls (*P <* 0.001; Fig. 3A). Alpha diversity in smoke-treated, γ-irradiated soils exhibited no difference between 10- and 35-days post-inundation but was significantly lower at 35-days post-inundation for the ambient controls (*P <* 0.0001; Fig. 3A).

NMDS analysis of the bacterial and archaeal community compositions revealed sample clustering based on the soil type, treatment, and incubation time factors (Fig. 3B) and were significantly different between the various levels of each factor based on PERMANOVA of the Bray-Curtis distances (*P <* 0.001). Significant interactions were also found between treatment and timepoint (*P <* 0.004), treatment and soil type (*P <* 0.001), and timepoint and soil type (*P <* 0.001). Multivariate data dispersion was homogeneous for the treatment factor, but the soil type and incubation time factors exhibited significant differences in multivariate dispersion (*P <* 0.01). No significant difference in dispersion was detected for the treatment factor, suggesting a significant effect of smoke treatment on community composition. While results suggest differences in community composition independent of the lower diversity of the irradiated soils, we cannot rule out the possibility that these differences may be due to a location effect. Nevertheless, the ordination supports a clustering pattern that confirms the importance of treatment on community composition of the soils, showing differences that continue along predictable trajectories as time-since-treatment increases (Fig. 3B).

**Figure 4:**
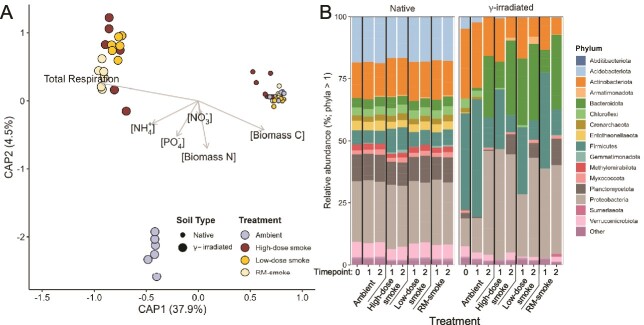
**Microbial species composition and drivers of community assembly.** (**A**) CAP ordination of community composition for bacterial and archaeal (16S rRNA genes) communities in soil mesocosms 10 days post-inundation constrained by soil environmental parameters. (**B**) Relative abundance of bacterial and archaeal (16S rRNA genes) phyla in soil mesocosms. Timepoint: 0 = time-zero controls; 1 = 10 days post-inundation; 2 = 35 days post-inundation.

Constrained ordination by CAP analysis was used to test the hypothesis that chemical composition of the soils correlates with community composition (Fig. 4A). Within the γ-irradiated soils at 10 days post-inundation, smoke-inundated samples form a cluster that is significantly different from ambient controls (*P <* 0.001; Fig. 4A). Overall, 42.4% of the variance in community composition is explained by CAP axes 1 and 2 and significant marginal effects (i.e., type III effects) of the chosen chemical drivers were observed for phosphate (*P <* 0.05), biomass C (*P <* 0.05), biomass N (*P <* 0.01), and total respiration (*P <* 0.001). At 35 days-post inundation, reflective of cumulative effects over time, significant marginal effects were also observed for pH (*P <* 0.001) and total respiration (*P <* 0.05; Supplementary Fig. A3A). Since native and γ-irradiated soils were inundated simultaneously from the same smoke source, CAP analysis constrained by PM concentrations in smoke was conducted separately for each soil type at 10 days post-inundation (Supplementary Fig. A3B-C). All three of the PM fractions (PM_10_, PM_2.5_, PM_1.0_) were observed to have significant marginal effects on the community composition in γ-irradiated soils (*P <* 0.001; Supplementary Fig. A3C); however, none were significant for the native soils (Supplementary Fig. A3B).

The relative abundance of bacterial and archaeal phyla remained relatively constant in the native soils regardless of treatment condition or incubation time (Fig. 4C). However, the γ-irradiated soils experienced much larger shifts in the relative abundance of specific taxa. Comparison of the time zero samples between soil types revealed that γ-irradiation alone increased the relative abundance of Actinobacteriota and Firmicutes from 14.3% and 5.7%, respectively in the native soils, to 28.1% and 39%, respectively in γ-irradiated soils. In contrast, Proteobacteria decreased from 24.4% to 11.1% (Supplementary Fig. A4). In the γ-irradiated ambient control soils, relative abundance of Firmicutes increased to 47.3% at timepoint 1 (10 days post-inundation), but sharply decreased to just 12.1% by timepoint 2 (35 days post-inundation; Supplementary Fig. A4). Conversely, Proteobacteria increased significantly to 42% by timepoint 2 (Supplementary Fig. A4). Smoke inundation enhanced the recovery of Proteobacteria for all tested smoke doses, increasing relative abundance to 44.5%, 25.2%, and 36.1% at timepoint 1 for the high-dose, low-dose, and RM-smoke treatments respectively, and reaching ~40% at timepoint 2 for all three smoke treatments (Supplementary Fig. A4). The Bacteroidota and Planctomycetota exhibited a similar trend, increasing in relative abundance over time for all treatments, while the Actinobacteria and Firmicutes did the opposite (Supplementary Fig. A4).

### Community composition of fuels and aerosols and source/sink relationships

**Figure 5:**
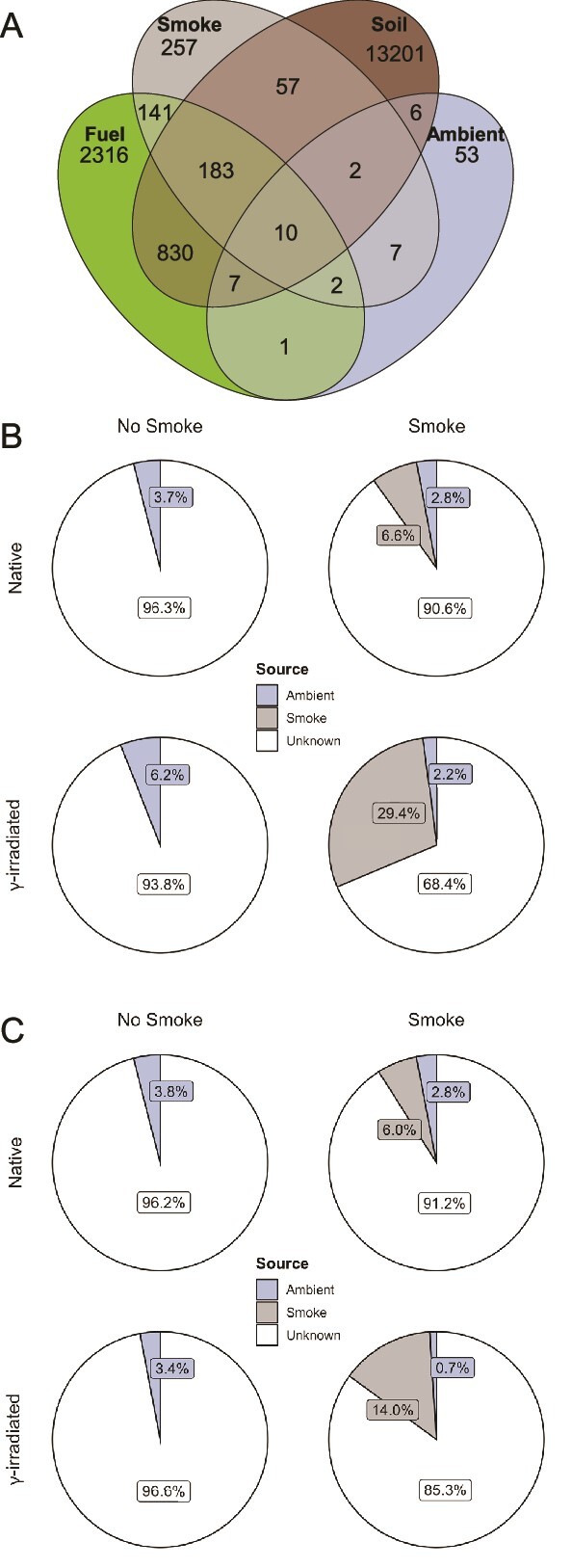
**Shared taxa between environmental compartments and source contribution to sink communities.** (**A**) Venn diagram of shared ASVs among fuel, smoke, ambient air, and soil samples. (**B**) Percent of ambient air/smoke sources contribution to native and γ-irradiated soil sinks 10 days post-inundation and (**C**) 35 days post-inundation based on FEAST analysis. “Unknown” source represents the portion of the community that could not be attributed to either of the known sources.

In total, 17 073 ASVs were observed in smoke-inundated soil, fuel, smoke, and ambient air samples (most to least ASVs; Fig. 5A). Smoke shared the most ASVs with fuel (51.0%) followed by soil (38.2%) and ambient air (3.2%; Fig. 5A). Soil shared 29.5% of fuel ASVs. Over 60% of ambient ASVs were restricted to this category, and in contrast, over 60% of smoke ASVs were shared with the other categories. The fuel microbiome was dominated by Proteobacteria (~50%), Bacteroidota (~25–30%), and Actinobacteriota (~20%; Supplementary Fig. A5A). The Proteobacteria were present in even larger proportions in the aerosol samples, representing ~70% of the ambient, ~75% of the high- and low-dose smoke, and ~90% of the RM-smoke communities (Supplementary Fig. A5A). NMDS analysis of the microbial communities revealed sample clustering by treatment at both the 10- and 35-days post-inundation timepoints, except for one high-dose and one low-dose smoke sample which clustered with the fuel samples (Supplementary Fig. A5B-C). PERMANOVA of the Bray-Curtis distances showed significant differences in community composition by compartment (*P <* 0.001); however, significant differences in dispersions (*P <* 0.05) were also detected.

When considering smoke as a source and soil as the sink, analysis with FEAST at 10 days post-inundation revealed ambient air contributed 3.7% of taxa in native control soils and double that (6.3%) in γ-irradiated control soils (Fig. 5B). Estimated source contributions at 35 days post-inundation remained nearly the same in the native control soils at 3.8% and decreased slightly in the γ-irradiated control soils to 3.4% (Fig. 5C). In contrast, at 10 days post-inundation smoke was attributed as the source for 6.6% and 29.4% of taxa in smoke-inundated native and γ-irradiated soils, respectively (Fig. 5B). Following a similar trend as the ambient control soils, the estimated source contribution of smoke at 35 days post-inundation was approximately the same in the native soils (6.0%) and decreased by roughly half in the γ-irradiated soils (14.0%; Fig. 5C).

## Discussion

### Hypothesis 1: smoke-inundated soil communities differ between native and sterilized soils

Significant differences in the composition of the soil communities were observed based on the treatment, soil type, and incubation time (Fig. 3B). The soil type and incubation time factors also exhibited significant differences in multivariate dispersion, indicating differences in composition may be due to location and/or dispersion effects for these factors. However, clear trends exist in the relative abundance of taxa based on all three factors (Fig. 4B). Actinobacteriota and Firmicutes were enriched by γ-irradiation alone, but the proportion of all remaining phyla decreased (Supplementary Fig. A4). Many members of the Firmicutes and Actinobacteriota are stress tolerant due to their ability to form spores and previous studies have also noted enrichment of these phyla in both irradiated soils [46, 47] and severely burned soils [31, 35, 48]. The bacterial and archaeal phyla in native soils did not exhibit major shifts in relative abundance regardless of the treatment or incubation time but were influenced by these factors to a greater extent in γ-irradiated soils (Fig. 4B). Consistent with our hypothesis, this indicates that differences in selective pressures exist between the two soil types due to competition between the native microbial community and those dispersed via smoke, which appears to limit the influence of smoke inundation on shifts in microbial abundances in undamaged soils. These trends are exemplified by the source tracking analysis that showed higher percentage contributions of smoke microbial communities in irradiated soils compared to native soils.

### Hypothesis 2: smoke-inoculated, sterilized soils have greater similarity to smoke assemblages than native soils, higher diversity than non-inundated sterilized soils, and lower genetic diversity than native soils

Dispersal influences microbial community reassembly following a disturbance in a variety of ecosystems [22, 49–54]. Likewise, the results of our source tracking analysis indicate that the smoke microbiome is the source of ~30% of the microbial community in smoke-treated, γ-irradiated soils at 10 days post-inundation compared to only 6.6% for native soils (Fig. 5B). Similarly, in a field-scale high-intensity wildland fire in a subalpine forest in Utah, USA, authors [17] used FEAST to quantify a 30% contribution of smoke to nearby soil sinks. Moreover, the proportions of Proteobacteria, Firmicutes, Bacteroidota, and Actinobacteriota in smoke-treated, γ-irradiated soils better reflect the proportions of these phyla in the fuel and smoke samples than do smoke-treated, native soils (Fig. 4B; Supplementary Fig. A5A). An experimental smoke treatment of soils [22] found a similar increase in the relative abundance of Proteobacteria in smoke-treated soils sampled 1 day after smoke-inundation. Although the authors did not track or test microbial transport from fuels through smoke into soils, their immediate post-smoke results indicate support for the conclusion that smoke dispersal contributes to the relative abundance of soil bacterial phyla following a major disturbance event [22].

Although the tallgrass prairie is fire-maintained, like other disturbances, wildland fire can reduce the abundance of susceptible taxa in burned surface soils, thereby decreasing soil microbial diversity, even if only temporarily [31, 55–57]. Consistent with our hypothesis, alpha diversity was significantly lower in γ-irradiated soils than native soils under all treatment conditions (Fig. 3A). Community diversity is influenced by both stochastic (e.g., ecological drift) and deterministic (e.g., selection via species competition) processes, the interplay of which determines the overall change in community composition. Dispersal is expected to increase the importance of selection by increasing the effective community size at the site of deposition [58], and the relative importance of drivers can change over time. Alpha diversity in smoke-treated, γ-irradiated soils remained approximately constant between 10- and 35-days post-inundation (Fig. 3A), suggesting that competition between the residual native microbial community and those introduced via smoke-deposition is the driving factor of microbial diversity in these soils. Contrary to our hypothesis, higher diversity was not evidenced between smoke-inundated and ambient exposed soils for either type of soil. The relative impact of different smoke dosages on beta diversity became more evident after 35 days (Fig. 3B), which could reflect stronger competition compared with RM-smoke, where fewer cells were dispersed into the soils. In contrast, the ambient, γ-irradiated soils exhibited a decrease in alpha diversity between the 10- and 35-day assessments (Fig. 3A) as well as increased distance from the initial clustering in the NMDS ordination (Fig. 3B), suggesting that ecological drift is enhanced when dispersal is low. Based on the spatial proximity of the clusters in γ-irradiated soils, high and low smoke treatment communities became more dissimilar from RM-smoke and ambient treatments as time progressed, suggesting that selection continues to play a role after the initial differentiating factor of smoke microbe dispersal.

### Hypothesis 3: the carbon efflux signal resulting from smoke inoculation is more easily detected in sterilized than in native soils, and higher “doses” of smoke result in increased soil respiration and impacts on soil physicochemical properties

Significant differences in soil respiration were found based on both the soil type and treatment factors (Fig. 1C-D). The use of native and γ-irradiated soils in these experiments was intended to simulate unburned and highly disturbed soils, respectively. Previous studies have reported that soil respiration decreases with increasing burn-severity [34, 59–61] and increasing doses of γ-radiation [62, 63]. Our results are seemingly in contrast with these observations, as total respiration in the first week after inundation was higher in γ-irradiated soils than native soils for all treatments (Fig. 1D). The difference in total respiration between soil types decreased by the end of the experiment, but remained higher in smoke-treated, γ-irradiated soils than native soils (Supplementary Fig. A1B). Smoke deposition is known to alter the nutrient content of soils [22] and microbial necromass following a disturbance event would similarly be expected to contribute to the free nutrient pool. Thus, the observed increases in respiration could be due to increased nutrient availability. However, treatment with RM-smoke from autoclaved fuels resulted in lower total respiration than treatment with high-dose smoke, despite burning the same amount of fuel in both conditions. This suggests that nutrient addition by smoke deposition and necromass alone is not the cause of the observed difference in soil respiration, but rather that viable microbes in smoke are dispersed in sufficient quantities to overcome the loss of biological activity from the initial sterilization. Moreover, the timing of peak respiration rates in γ-irradiated soils also depended on the treatment, with those dispersing more viable cells (high- and low-dose smoke; Table 1) exhibiting peak respiration rates earlier than those which dispersed fewer viable cells (RM-smoke and ambient; Table 1, Fig. 1C).

Although soil respiration in the first week after inundation differed between treatments, microbial biomass did not. Significant differences in microbial biomass C were only observed between soil types, with smoke-treated, γ-irradiated soils having less biomass C than native soils as would be expected after sterilization (Fig. 2A). Microbial biomass N was only found to be significantly lower for γ-irradiated soils treated with low-dose smoke (Fig. 2B). Microbial growth and activity in soil is influenced by soil chemistry, especially the concentration of C and N pools [64–66]. Gamma irradiation is generally considered to be the least disruptive method of soil sterilization, with less impact on soil physicochemical properties [67–69]. Nevertheless, notable changes in specific chemical properties following irradiation have consistently been observed. At doses between 30–50 kGy, as was used in this study, dissolved organic carbon and ammonium concentrations increase [63, 67, 70–72], likely due to the lysis of microbial cells [73]. Our results agree with these observations, as TOC was significantly higher in γ-irradiated soils (Supplementary Fig. A2A) and ammonium tended to be higher in these soils as well, though not statistically significant (Supplementary Fig. A2C). Within this same dose range, TN appears to remain unchanged [74], whereas reports of any effects on pH have shown increase, decrease, and no significant change [67, 69, 75]. TN in our γ-irradiated soils tended to be higher than native soils, though this difference was only significant for the ambient controls and those treated with RM-smoke (Supplementary Fig. A2B). By the end of the 35-day experiment, soil pH was significantly higher in γ-irradiated soils than native soils for all treatments (Fig. 2D).

Changes in soil physicochemical properties following wildland fire are also known to influence microbial community structure [22, 76–81]. In our study, we observed significant marginal effects on community composition in γ-irradiated soils for phosphate, biomass C and N, total respiration (Fig. 4A), and pH (Supplementary Fig. A3A). In addition, we observed shifts in the relative abundance of soil microbial phyla depending on the treatment received. Though initially enriched by γ-irradiation, the proportions of Firmicutes and Actinobacteriota in the irradiated, ambient control soils decreased by 35 days post-inundation to levels approximately equal to those observed in the native soils, although proportions of Proteobacteria and Bacteroidota significantly increased over time (Fig. 4B). Ferrenberg et al. (2013) observed a similar decrease in Actinobacteriota and increase in Proteobacteria (specifically Betaproteobacteria) with increased time since burning, and others have noted increases in Actinobacteriota, Proteobacteria, and Bacteroidota in the months following a severe wildfire event [57, 82]. In our study, the recovery of Proteobacteria was enhanced by smoke inundation in a dose-dependent manner. Treatment of γ-irradiated soils with higher smoke doses increased the proportion of Proteobacteria at 10 days post-inundation to approximately the same level seen in the ambient controls at 35 days post-inundation (Fig. 4B). Bacteroidota proportions were better enhanced by treatment with low-dose smoke, though the high-dose and RM-smoke treatments also improved the recovery of this phylum relative to ambient controls (Fig. 4B). Taken together, these results suggest that the dispersal of microbes by smoke after a major disturbance event influences the composition of the successive microbial community.

Our study simulated smoke dispersal during wildland fires using controlled laboratory burn experiments and evaluated the effects on microbial community assembly, respiration, and composition over time. The effect of the higher dose of smoke on respiration rates in native soils did result in a temporary spike in activity, but the long-term response was muted. Following smoke-inundation, respiration rates were markedly higher in γ-irradiated soils than native soils for all smoke levels, with peak respiration rates occurring earlier in the high- and low-dose smoke conditions characterized by higher concentrations of viable cells than the other treatments. The enhanced microbial activity in the first week of incubation was likely a result of smoke-driven dispersal coupled with decreased competition due to the inactivation of many cells by pretreatment with γ-radiation. In addition, the necromass resulting from these inactivated cells likely contributed to the higher nutrient content observed in γ-irradiated soils, presenting the opportunity for smoke-dispersed microbes to successfully colonize the deposition site, whereas the established community in native soils appeared to reduce the impact of such settlers. These experimental smoke treatments were limited to 10–20 min and reflected a very small mass of fuel consumed; on the order of grams vs. the hundreds of megagrams per hectare consumed in typical wildfires [25]. The impacts of longer-duration or higher-dosage smoke dispersal from fuel consumption more reflective of *in situ* wildland fire may differ from these results in both quantity and quality, and in response to complex atmospheric processes affecting aerosolized microbes and the physicochemical impacts of longer-duration smoke on soil microbial processes.

## Supplementary Material

ISME_Supplemental_Information_Inundation_Revision_1_LK_wrae203

## Data Availability

Sequence data generated from this work are deposited and available in the NCBI database under the BioProject number PRJNA1140635.
