# Correction to: Dispersal of microbes from grassland fire smoke to soils

**DOI:** 10.1093/ismejo/wrae232

**Published:** 2024-12-04

This is a **correction** to:

Adam J Ellington, Kendra Walters, Brent C Christner, Sam Fox, Krista Bonfantine, Cassie Walker, Phinehas Lampman, David C Vuono, Michael Strickland, Katie Lambert, Leda N Kobziar, Dispersal of microbes from grassland fire smoke to soils, *The ISME Journal*, Volume 18, Issue 1, January 2024, wrae203, https://doi.org/10.1093/ismejo/wrae203

In the originally published version of this manuscript, the funding sources were inadvertently noted in the `Acknowledgements' section rather than the `Funding' section. This error has been corrected.

