# Supplementary material for: Dispersal of microbes from grassland fire smoke to soils
Source: ISME J. 2024 Oct 15;18(1):wrae203. doi: 10.1093/ismejo/wrae203 (PMC11525542; doi:10.1093/ismejo/wrae203)
Supplement: ISME_Supplemental_Information_Inundation_Revision_1_LK_wrae203 [file isme_supplemental_information_inundation_revision_1_lk_wrae203.docx]

**Appendix A:** Supplemental Information for: Dispersal of microbes from grassland fire smoke to soils

Adam J. Ellington, Kendra Walters, Brent C. Christner, Sam Fox, Krista Bonfantine, Cassie Walker, Phinehas Lampman, David Vuono, Michael Strickland, Katie Lambert, Leda N. Kobziar

**This section includes:**

# Supplemental Materials and Methods

# Supplemental Figures A1-A5

Supplemental Materials and Methods

*DNA Extraction, Amplification, and Sequencing*

Dried fuels were ground using an electric coffee grinder (Amazon Basics), followed by bead beating for 5 min at maximum speed using a BeadBug Microtube Homogenizer (Benchmark Scientific, Sayreville, New Jersey, USA). Debris was removed by centrifugation and the supernatant was transferred to the Zymo-Spin™ III-F Filter in a collection tube. An additional 500 μL of lysate buffer was added to the remaining debris and samples were vortexed at maximum speed for 30 sec (Fisher Scientific, Waltham, Massachusetts, USA). Following centrifugation, the supernatants were recombined, and the manufacturer’s recommended protocol was continued unmodified. Extracted DNA was quantified with a ND2000 spectrophotometer (NanoDrop Technology, Wilmington, Delaware) and standardized to 5 ng/μL prior to PCR amplification.

Samples were bead beaten at max speed for 5 minutes using a BeadBug (Benchmark Scientific, Sayreville, New Jersey, USA), and all supernatant (instead of 400 μL) was transferred to the collection tube. Following elution, the eluate was returned to the column and centrifuged a second time to collect additional DNA from the filter. These modifications were made due to the low biomass and DNA yield of these samples. Extracted DNA was cleaned and concentrated using the Zymo DNA Clean and Concentrator–5 kit (Zymo Research, Irvine, California, USA) with a final elution volume of 10 μL.

For all samples, primers targeting the bacterial/archaeal ribosomal RNA gene (16S) were used based on the Earth Microbiome Project’s primer recommendations (primers 515F & 806R). Reactions for amplification were mixed as follows: 1 μL sample DNA, 12.5 μL 2X Phusion Plus MasterMix (ThermoFisher Scientific, Pittsburg, PA) and 1.5 μL each forward and reverse primers for a total reaction volume of 25 μL. Fuel and soil samples were amplified in triplicate according to the manufacturer’s recommended protocol, with 25 cycles of amplification. Due to low DNA yield, the air and smoke samples were amplified in singlet with 35 cycles of amplification.

Following amplification, each set of triplicate reactions were combined and cleaned using the MagBio HighPrep PCR Clean-up kit (MagBio Genomics Inc., Gaithersburg, MD) following the manufacturer’s protocol except that a 1:1 ratio of bead solution to PCR volume and a third wash step with 80% EtOH were also used. After PCR clean up, 5uL of the air samples, 1uL of soil samples, and 2uL of fuels samples were subjected to a second PCR of 10 cycles using primers to attach index sequences and adaptors for Illumina sequencing. Amplicons were then visualized by agarose gel electrophoresis to verify amplification and cleaned again using the MagBio HighPrep PCR Clean-up kit as described above. DNA was quantified using a Qubit 2.0 fluorometer and the Qubit dsDNA HS Assay kit (ThermoFisher Scientific, Pittsburg, PA). All samples were pooled based on sample type to a total of 50 ng of DNA per sample and sequenced using the MiSeq (Illumina) platform at the Genomics and Bioinformatics Resources Core at the University of Idaho.

*Bioinformatics and Statistical Analysis*

Each sequencing run was processed separately to independently calculate error rates and infer amplicon sequence variants (ASVs). Reads were trimmed to 240 bp (forward) and 150 bp (reverse) before applying default quality filtering variables with no ambiguous bases allowed and a minimum length of 50bp. Reads were then dereplicated, pairs were merged, and ASVs were inferred. Differences in the abundance of individual taxa within positive controls was assessed using the emmeans function (v.1.8.2; 1) and sequencing runs were merged after no significant differences were detected between runs. Chimeras were removed from the appended sequence table before taxonomic assignment was performed against the Silva 138.1 database (2) using the ‘assignTaxonomy’ function with a minimum bootstrap of 80, followed by ‘addSpecies’ to append species designations where possible. The phyloseq object constructed from the DADA2 output was filtered using ‘subset_taxa’ (v.1.40.0; McMurdie & Holmes, 2013) to remove sequences attributed to Eukaryota, chloroplasts, or mitochondria. Taxa lacking an assignment at the Kingdom level were removed but taxa that were unassigned below Kingdom were retained. Putative contaminant taxa were identified using ‘decontam 1.16.0’ (4) in a two-step process using the prevalence method with a default threshold of 0.1. In the first step, lab blanks were compared to all samples within each sequencing run. Putative lab contaminants were removed before a second round of decontamination was performed on aerosol samples with the appropriate field blanks for each experiment and day (if applicable).

ASV counts were normalized to proportions (5) and a square-root transformation was applied to reduce the influence of dominant taxa in the Bray-Curtis distance matrix. Significant differences in community composition between levels of the soil type, treatment, and incubation time factors were determined through distance-based permutational multivariate analysis of variance (PERMANOVA) using the adonis2 function of the vegan package with 999 permutations. Homogeneity of dispersion between levels of the soil type, treatment, and incubation time factors was assessed using the permutest and betadisper functions of the vegan package. Canonical analysis of principal coordinates (CAP; Anderson & Willis, 2003) was used to test whether soil chemical composition correlates with microbial community composition. Significance for the marginal effects of each environmental parameter was assessed by ANOVA-like permutation testing using the anova.cca function of the vegan package. ASVs shared between the fuel, smoke, and soil sample types were weighted by relative abundance and visualized using the ps_venn function of the MicEco package.

Analyses were conducted in the R environment. Differences in total respiration, chemical composition, and pH between samples were tested for normality using the Shapiro-Wilk test. Analysis of variance (ANOVA) was used to test for individual and interacting effects between the soil type and treatment groups on the response variable, followed by multiple pairwise comparisons within each group using Tukey’s post-test. In the case of non-normal distributions, the Kruskal-Wallis test was used to test the simple main effects only, followed by pairwise comparisons using Dunn’s test. Welch’s ANOVA was used where data was normally distributed but had unequal variances between test groups. Significance was set at the *P* < 0.05 level unless otherwise noted.

References

1. Lenth R, Buerkner P, Herve M, Jung M, Love J, Miguez F. emmeans: estimated marginal means, aka least-squares means. Version 1.8. 2: CRAN, R. 2022.

2. McLaren MR, Callahan BJ. Silva 138.1 prokaryotic SSU taxonomic training data formatted for DADA2 [Internet]. Zenodo; 2021 [cited 2023 Aug 17]. Available from: https://zenodo.org/record/4587955

3. McMurdie PJ, Holmes S. phyloseq: An R Package for Reproducible Interactive Analysis and Graphics of Microbiome Census Data. PLOS ONE. 2013 Apr 22;8(4):e61217.

4. Davis NM, Proctor DM, Holmes SP, Relman DA, Callahan BJ. Simple statistical identification and removal of contaminant sequences in marker-gene and metagenomics data. Microbiome. 2018 Dec 17;6(1):226.

5. Anderson MJ, Willis TJ. Canonical Analysis of Principal Coordinates: A Useful Method of Constrained Ordination for Ecology. Ecology. 2003;84(2):511–25.

**
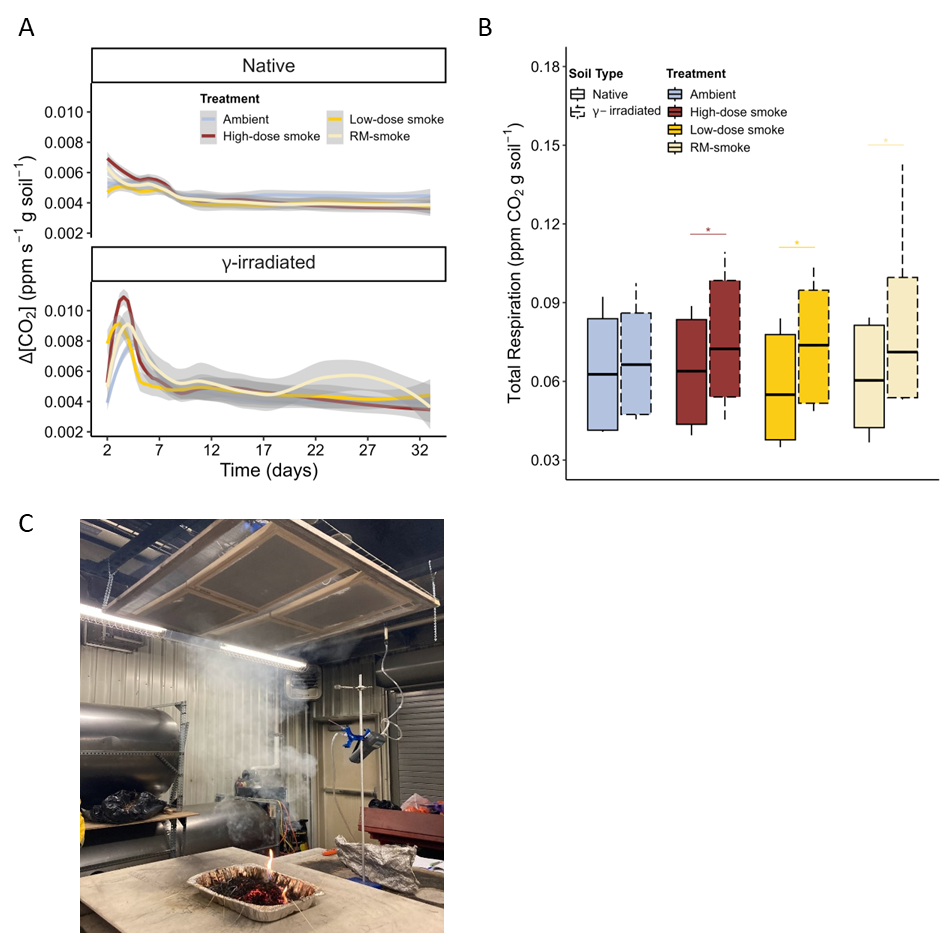
**

**Figure A1: Effect of smoke inundation on soil respiration. A)** Soil respiration rates measured as Δ[CO_2_] (ppm s-1 g soil-1) over 32 days of incubation. Grey area around each line represents 95% confidence interval. **B)** Sum of the respiration rates for all 32 days of incubation. Boxes represent the interquartile range, the middle horizontal line is the median, and the whiskers represent minimum and maximum values. Asterisks represent significance (*

= p < 0.05; Dunn’s test). **C)** Photo showing soils suspended above burning prairie grassland fuels during inundation experiment; particulate matter samplers are also located on suspended canopy above the burn (University of Idaho IFIRE Combustion Lab; K. Walters, photo).


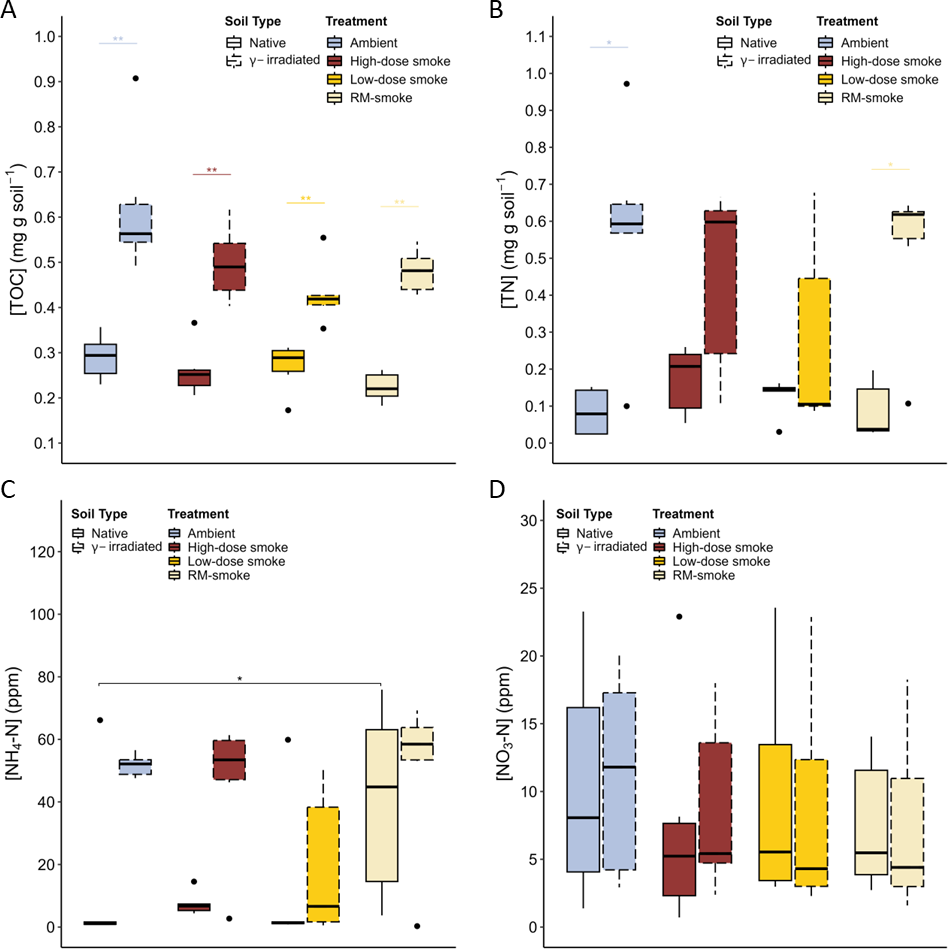


**Figure A2: Effect of smoke inundation on the chemical composition of soils.** Concentrations of **A)** TOC, **B)** TN, **C)** ammonium, and **D)** nitrate for samples taken 10 days post-inundation. Boxes represent the interquartile range, the middle horizontal line is the median, and the whiskers represent minimum and maximum values. Outliers are 1.5 interquartile ranges below the first quartile and above the third quartile. Asterisks represent significance (* = p < 0.05; ** = p < 0.01; Kruskal-Wallis and Dunn’s test).


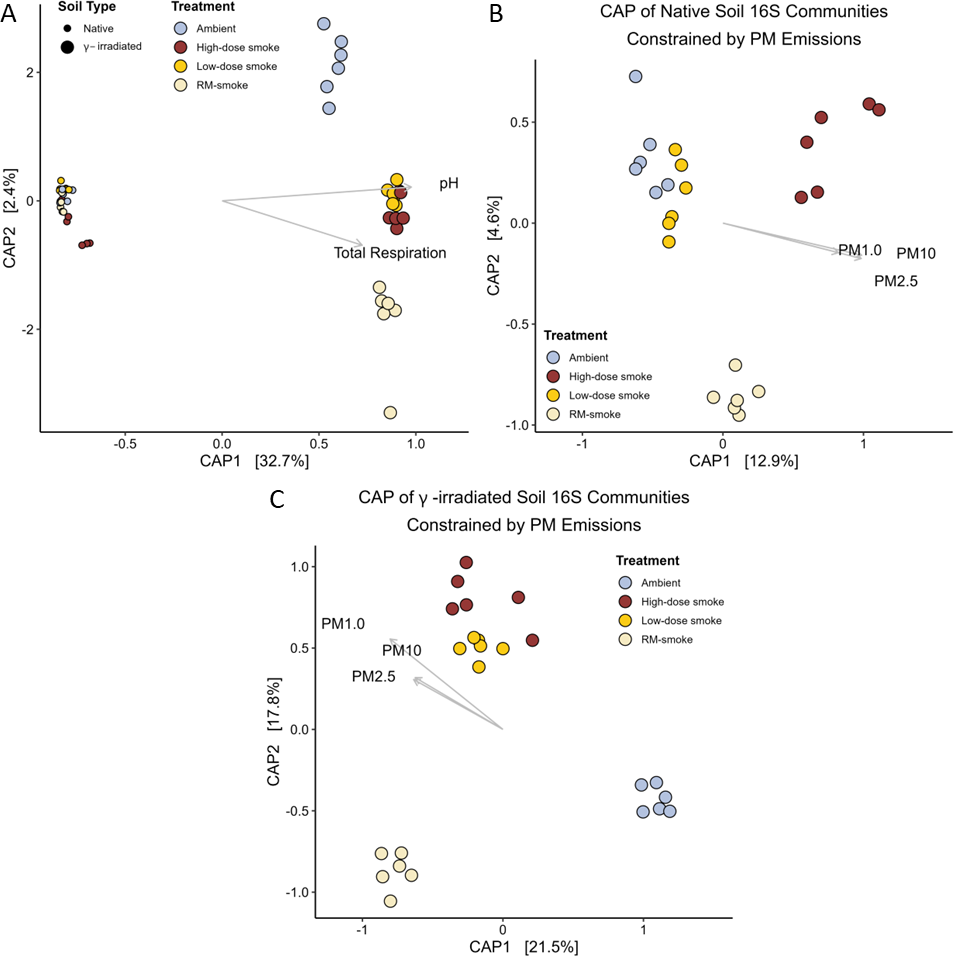


**Figure A3: Drivers of community assembly within native and γ-irradiated soils. A)** CAP ordination of community composition for 16S communities in soil mesocosms 35 days post-inundation constrained by soil environmental parameters. CAP ordination of community composition in **B)** native and **C)** γ-irradiated soils for 16S communities 10 days post-inundation constrained by PM emission concentrations.


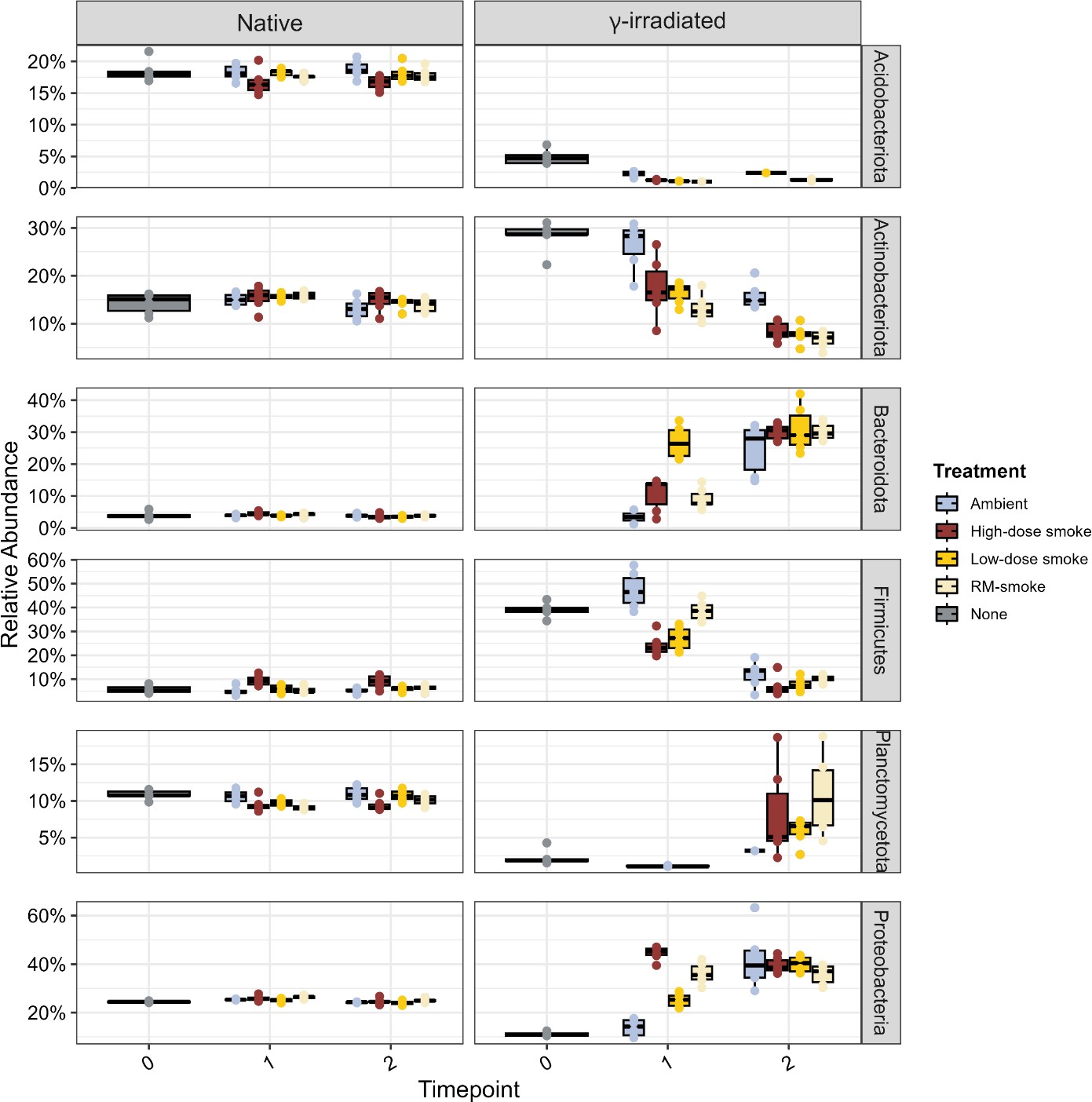


**Figure A4: Relative abundance of select phyla in soil mesocosms.** Timepoint: 0 = time-zero controls; 1 = 10

days post-inundation; 2 = 35 days post-inundation.


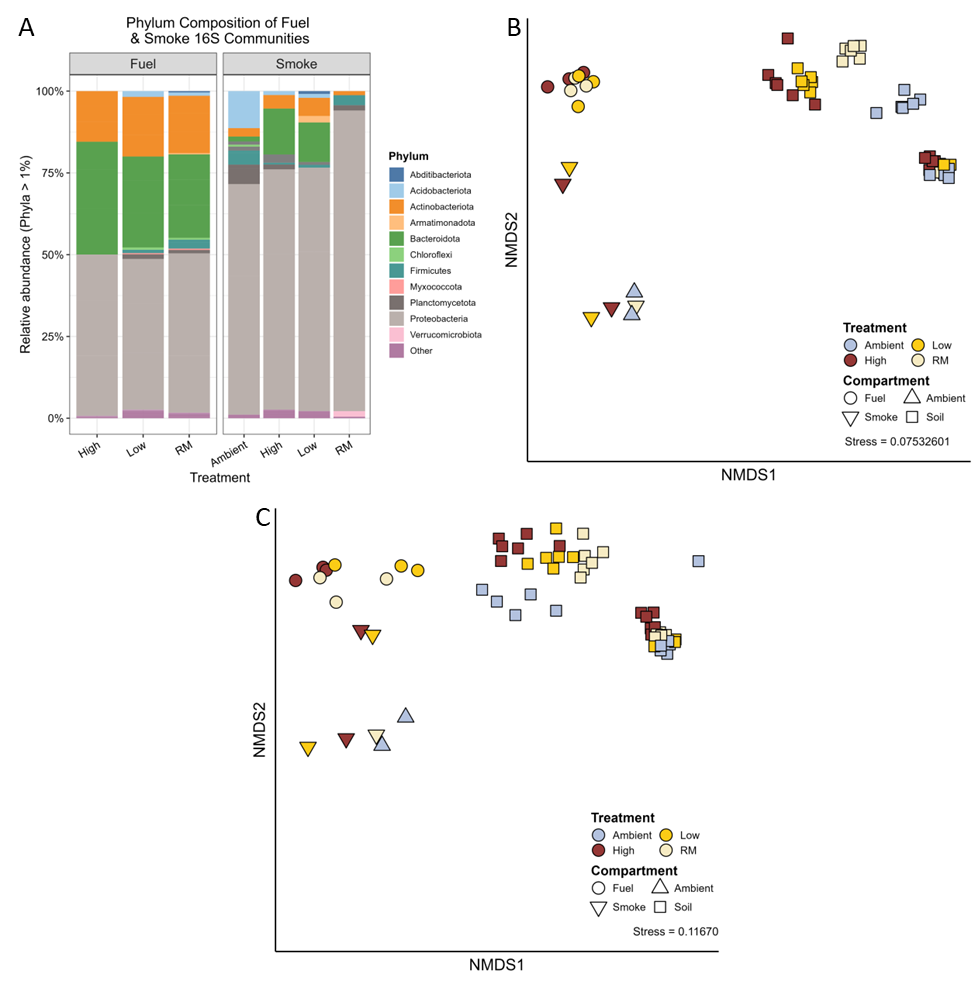


**Figure A5: Microbial community composition of fuel and smoke samples. A)** Relative abundance of bacterial and archaeal (16S) phyla in the fuel and aerosol samples. NMDS ordination of community composition by treatment and compartment for bacterial and archaeal (16S) communities in soil mesocosms taken at **B)** 10 days post-inundation and **C)** 35 days post-inundation.
